# Early Outcomes of Two Large Mitral Valve Transcatheter Edge-to-Edge Repair Devices—A Propensity Score Matched Multicenter Comparison

**DOI:** 10.3390/jcm13144187

**Published:** 2024-07-17

**Authors:** Philipp von Stein, Hendrik Wienemann, Jennifer von Stein, Atsushi Sugiura, Tetsu Tanaka, Refik Kavsur, Can Öztürk, Marcel Weber, Jean Marc Haurand, Patrick Horn, Tobias Kister, Amir Abbas Mahabadi, Niklas Boeder, Tobias Ruf, Muhammed Gerçek, Christoph Mues, Christina Grothusen, Julia Novotny, Ludwig Weckbach, Henning Guthoff, Felix Rudolph, Amin Polzin, Stephan Baldus, Tienush Rassaf, Holger Thiele, Helge Möllmann, Malte Kelm, Volker Rudolph, Ralph Stephan von Bardeleben, Holger Nef, Peter Luedike, Philipp Lurz, Jörg Hausleiter, Roman Pfister, Victor Mauri

**Affiliations:** 1 Department of Cardiology, Heart Center, Faculty of Medicine, University of Cologne, 50937 Cologne, Germany; 2 Department of Internal Medicine II, Heart Center Bonn, University Hospital Bonn, 53127 Bonn, Germany; 3 Department of Cardiology, Pulmonology and Vascular Medicine, University Hospital Düsseldorf, Medical Faculty of the Heinrich Heine University Düsseldorf, 40225 Düsseldorf, Germanyamin.polzin@med.uni-duesseldorf.de (A.P.); malte.kelm@med.uni-duesseldorf.de (M.K.); 4 Department of Cardiology, Heart Center Leipzig at University of Leipzig, 04289 Leipzig, Germany; 5 Department of Cardiology and Vascular Medicine, West German Heart and Vascular Center, University Hospital Essen, 45122 Essen, Germany; 6 Department of Cardiology and Angiology, Justus-Liebig-University Giessen, 35392 Giessen, Germany; 7 Department of Cardiology, University Medical Center of the Johannes Gutenberg-University Mainz, 55131 Mainz, Germany; 8 Clinic for General and Interventional Cardiology/Angiology, Herz- und Diabeteszentrum NRW, Ruhr-Universität Bochum, 32545 Bad Oeynhausen, Germany; 9 Medical Clinic I, Department of Cardiology, St. Johannes Hospital, 44137 Dortmund, Germany; 10 Department of Cardiovascular Surgery, University Hospital Schleswig-Holstein, Campus Kiel, 24105 Kiel, Germany; 11 Medizinische Klinik und Poliklinik I, Ludwig-Maximilians-Universität München, 81377 Munich, Germany

**Keywords:** PASCAL, MitraClip, mitral regurgitation, mitral valve transcatheter edge-to-edge repair

## Abstract

**Background/Objectives:** Previous trials reported comparable results with PASCAL and earlier MitraClip generations. Limited comparative data exist for more contemporary MitraClip generations, particularly the large MitraClip XT(R/W). We aimed to evaluate acute and 30-day outcomes in patients undergoing mitral valve transcatheter edge-to-edge repair (M-TEER) with one of the large devices, either PASCAL P10 or MitraClip XT(R/W) (3rd/4th generation). **Methods:** A total of 309 PASCAL-treated patients were matched by propensity score to 253 MitraClip-treated patients, resulting in 200 adequately balanced pairs. Procedural, clinical, and echocardiographic outcomes were collected for up to 30 days, including subgroup analysis for mitral regurgitation (MR) etiologies. **Results**: PASCAL and MitraClip patients were comparable regarding age (80 vs. 79 years), sex (female: 45.5% vs. 50.5%), and MR etiology (degenerative MR: n = 94, functional MR [FMR]: n = 96, mixed MR: n = 10 in each group). Technical success rates were comparable (96.5% vs. 96.0%; *p* > 0.999). At discharge, the mean gradient was higher (3.3 mmHg vs. 3.0 mmHg; *p* = 0.038), and the residual mitral valve orifice area was smaller in MitraClip patients (3.0 cm^2^ vs. 2.3 cm^2^; *p* < 0.001). At discharge, the reduction to MR ≤ 2+ was comparable (92.4% vs. 87.8%; *p* = 0.132). However, reduction to MR ≤ 1+ was more frequently observed in PASCAL patients (67.7% vs. 56.6%; *p* = 0.029), driven by the FMR subgroup (74.0% vs. 60.0%; *p* = 0.046). No difference was observed in 30-day mortality (*p* = 0.204) or reduction in NYHA-FC to ≤II (*p* > 0.999). **Conclusions**: Both M-TEER devices exhibited high and comparable rates of technical success and MR reduction to ≤2+. PASCAL may be advantageous in achieving MR reduction to ≤1+ in patients with FMR.

## 1. Introduction

Mitral valve transcatheter edge-to-edge repair (M-TEER) has emerged as a safe, effective, and most commonly used alternative to mitral valve (MV) surgery for the treatment of mitral regurgitation (MR) in patients at increased surgical risk [1]. Two M-TEER systems, the PASCAL (Edwards Lifesciences, Irvine, CA, USA) and the MitraClip (Abbott Structural Heart, Santa Clara, CA, USA) systems, are currently available in Europe and the United States. However, these systems employ distinct mechanisms to achieve MV leaflet approximation, particularly in terms of device design, closure mechanism, maneuvering, and grasping.

The more recently introduced PASCAL P10 has several technical features aimed at reducing stress on the MV leaflets, including an atraumatic grasping feature and broad, contoured paddles. To date, studies on the PASCAL system have primarily been performed on anatomically and clinically highly selected patients [2,3]. The MitraClip system, on the other hand, has been used in clinical practice for many years, with over 180,000 patients treated to date [4]. A larger MitraClip device with longer arms and grippers, the MitraClip XTR, was introduced for the first time with the 3rd generation (G3) MitraClip and enabled the treatment of a broader range of MV pathologies [4,5]. The 4th generation (G4) MitraClip introduced wider clip arms to minimize stress on the leaflets and continued devices with longer arms and grippers (XT and XTW). Once again, encouraging short-term results were observed in the global, prospective, multicenter, single-arm EXPAND G4 observational study [4,6].

Although several studies have compared the PASCAL with the MitraClip, these studies were limited in terms of MR etiology, confined to earlier generations of the MitraClip, and/or included the smaller MitraClip NT (NTR/NT/NTW), limiting these comparisons as smaller devices may be employed for different MV pathologies than larger devices [2,7,8,9,10,11,12]. Direct comparisons between the equally sized PASCAL P10 and MitraClip XT(R/W) are lacking. In this study, we evaluated patients from the largest PASCAL post-approval cohort against those treated with the MitraClip XT(R/W) [11,13]. 

## 2. Materials and Methods

### 2.1. Study Population and Data Collection

This retrospective multi-center study comprises two cohorts treated for symptomatic moderate-to-severe or severe MR:

(1) The PASCAL cohort includes 309 consecutive patients treated with the PASCAL P10 (Edwards Lifesciences, Irvine, CA, USA) between February and December 2019 at 10 tertiary care centers in Germany, and the results of this cohort have previously been published [13].
(2) The MitraClip cohort includes 253 consecutive patients treated with the MitraClip XT(R/W) (Abbott Structural Heart, Santa Clara, CA, USA) between May 2018 and April 2022 at 3 of the 10 centers (University Hospitals in Bonn, Cologne, and Duesseldorf).

Prior to M-TEER, all patients underwent comprehensive clinical and echocardiographic evaluations and were deemed unsuitable for surgery by the respective local interdisciplinary heart team, based not only on the EuroSCORE II but on other factors that may influence surgical risk, such as liver dysfunction, porcelain aorta, obesity, low platelet count, and frailty [14]. Both cohorts did not have any pre-specified inclusion or exclusion criteria. However, the first four PASCAL-treated patients at each center were selected in collaboration with the manufacturer [13]. The decision to use either the PASCAL or MitraClip was at the discretion of local physicians, which also depended on availability, as the G3 MitraClip XT (XTR), G4 MitraClip XT (XT and XTW), and PASCAL P10 devices received Conformité Européene approval in 2018, 2020, and 2019, respectively. The respective local ethics committees approved the retrospective data collection and waived the requirement for informed consent from subjects. The anonymized data were analyzed centrally.

### 2.2. Procedures

The PASCAL and MitraClip procedures were performed under the guidance of transesophageal echocardiography and fluoroscopy. The decision to use general anesthesia or deep sedation was made by the local physicians. Detailed descriptions of the procedure for the PASCAL and MitraClip devices have been published earlier [3,15]. The P10 device was used for every PASCAL procedure, and all MitraClip procedures utilized an XT(R/W) device; accordingly, only G3 and G4 MitraClips were used.

### 2.3. Echocardiographic Assessment

Echocardiographic assessment was performed at baseline, discharge, and 30-day follow-up. The severity of MR was graded based on a multiparametric approach, in line with current guidelines recommendations as follows: No MR (0), mild (1+), mild-to-moderate (2+), moderate-to-severe (3+), and severe (4+) [16,17]. Three echo experts from three centers (University Hospitals in Bonn, Cologne, and Duesseldorf) have been reviewing 20% of randomly selected patients from the PASCAL cohort, and excellent inter-observer agreement was observed [11]. Echocardiographic outcomes in the MitraClip cohort have been adjudicated by the same experts from the three centers providing MitraClip data.

### 2.4. Endpoints and Follow-Up

Local investigators evaluated clinical and echocardiographic outcomes, and 30-day follow-up appointments were scheduled as part of routine clinical practice. Outcomes were based on the consensus of the Mitral Valve Academic Research Consortium (MVARC), except when specifically stated [18]. The primary endpoints included technical success and MR severity at discharge. Additionally, instances of procedural damage to the native MV apparatus (defined as damage to the chords, papillary muscles, leaflets, or the mitral annulus) were collected [17]. Secondary endpoints encompassed severity of MR at 30 days, sustainability of M-TEER (paired comparison of MR ≤ 1+/2+ at discharge and 30 days), and device success at 30 days as defined by the MVARC (successful device implantation, MR ≤ 2+, mean transmitral gradient < 5 mm Hg, and absence of mortality, stroke, unplanned surgical/interventional procedures, and device failure) [18]. The 30-day clinical endpoints comprised all-cause mortality, severe bleeding, stroke, renal failure requiring dialysis, reintervention for MV dysfunction, and New York Heart Association Functional Class (NYHA-FC) [18]. 

### 2.5. Statistical Analysis

Continuous variables were presented as mean ± SD or median (Q1–Q3), as appropriate. The distribution of continuous variables was assessed with the Shapiro–Wilk test. Categorical variables are expressed as frequencies and percentages. Unpaired testing was performed using the Fisher’s exact test for categorical data, the Mann–Whitney U test, or the Student’s *t*-test, as appropriate. Paired testing was conducted using a paired Student’s *t*-test, a Wilcoxon signed-rank test, or a McNemar test, as appropriate. The 30-day mortality was assessed using Kaplan-Meier analysis (Log-rank test). To adjust for variations in patient characteristics at baseline, propensity score (PS) matching was used to identify similar cohorts of patients treated with the PASCAL P10 and the MitraClip XT(R/W). PS matching was performed individually for each MR etiology (degenerative MR [DMR], functional MR [FMR], and mixed MR [MMR]). The PS included sex, age, NYHA-FC, left ventricular ejection fraction (LVEF), left ventricular end-diastolic diameter (LVEDD), left atrial volume index (LAVI), effective regurgitant orifice area (EROA), and mean transmitral gradient. Missing values for variables used for the PS were addressed using 5-fold multiple imputations. We applied a nearest neighbor matching algorithm in a 1:1 ratio without replacement, using a 0.2 caliper setting. Standardized mean differences, with values < 0.2 considered as no meaningful imbalance, were calculated to examine the balance before and after matching. In a sensitivity analysis, we compared MR reduction to ≤ 2+ and ≤ 1+ at discharge and 30-days in patients treated with PASCAL to patients treated with MitraClip G3 and separately to those treated with MitraClip G4. In addition, these parameters were compared between patients treated with MitraClip G3 and G4. Two-sided p-values below 0.05 were considered statistically significant. Statistical data analyses were conducted using IBM SPSS Statistics version 28.0 (IBM, Armonk, NY, USA) and R version 4.3.1 (R Foundation for Statistical Computing, Vienna, Austria) with the MatchIt Package extension.

## 3. Results

### 3.1. Study Populations and Baseline Characteristics

Unmatched clinical and echocardiographic characteristics as well as procedural and follow-up outcomes of the PASCAL cohort have been published previously and are provided in Supplemental Tables S1–S4 for the MitraClip cohort, respectively [13].

PS matching resulted in 200 adequately balanced pairs, which were included in subsequent analyses (Graphic Abstract, Supplemental Figure S1). Table 1 presents the baseline characteristics of the matched cohorts. Median age (PASCAL: 80 years; MitraClip: 79 years), proportion of females (PASCAL: 45.5%; MitraClip: 50.5%), and distribution of MR etiologies (DMR: n = 94, FMR: n = 96, MMR: n = 10 in each group) were comparable, as were all other baseline characteristics except for arterial hypertension, diabetes mellitus, and chronic lung disease. Supplemental Table S5 provides clinical baseline characteristics categorized by MR etiology after matching. Based on this categorization, PASCAL-treated DMR patients had a higher EuroSCORE II and a higher prevalence of diabetes mellitus. 

Echocardiographic baseline parameters are reported in Table 2 and, categorized by MR etiology, in Supplemental Table S6. Matching resulted in an adequate balance of echocardiographic parameters without significant differences, which also applied to the subgroups.

### 3.2. Procedural and In-Hospital Outcomes

Procedural details and in-hospital outcomes are shown in Table 3. All PASCAL patients were exclusively treated with the PASCAL P10 device. MitraClip patients were treated with G3 (XTR implanted in 75.0%) or G4 (XT and/or XTW implanted in 22.5%) devices. One patient (0.5%) was treated with both one MitraClip XTR (G3) and one MitraClip XT (G4). Additional secondary devices (NTR, NT, and NTW) were implanted in MitraClip patients, with the MitraClip NTR being the most commonly used device. Details of the device selection for MitraClip patients are presented in Supplemental Table S7. MitraClip patients received significantly more devices than PASCAL patients (PASCAL: 1.3 ± 0.4 vs. MitraClip: 1.6 ± 0.6; *p* < 0.001). A total of 142 (71.0%) PASCAL patients and 84 (42%) MitraClip patients received one device. Two devices were implanted in 53 (26.5%) PASCAL patients and 98 (49.0%) MitraClip patients. No PASCAL patients received three devices, while 14 (7.0%) MitraClip patients received three devices (all *p* < 0.001).

The median procedure duration was 83 and 86 minutes for PASCAL and MitraClip patients, respectively (*p* = 0.783). Three percent (n = 6) of MitraClip patients underwent concomitant tricuspid valve intervention, whereas no PASCAL patients received such intervention (*p* = 0.031). Data on independent grasping were available for 181 PASCAL and 46 MitraClip G4 patients. Independent leaflet grasping was employed in 58.0% (105 out of 181) of PASCAL patients and 26.0% (12 out of 46) of MitraClip patients (*p* < 0.001). Technical success rates were equally high in both PASCAL and MitraClip patients (96.5% vs. 96.0%; *p* > 0.999). In the PASCAL group, technical success was not achieved in a total of 7 patients (3.5%). Among these, the procedure was aborted in 5 patients (2.5%), and 2 patients (1.0%) experienced an intraprocedural single leaflet device attachment (SLDA). Technical success was not achieved in 8 patients (4.0%) within the MitraClip group. The procedure was aborted in 4 out of these patients (2.0%), including 1 patient who suffered damage to the native MV apparatus. Two patients (1.0%) experienced intraprocedural SLDAs, with one additionally experiencing damage to the native MV apparatus. One patient (0.5%) required conversion to MV surgery due to damage to the native MV apparatus, while another patient suffered this incident only. Overall, damage to the native MV apparatus occurred exclusively in MitraClip patients (4 out of 200) and occurred with equal frequency in both DMR (n = 2) and FMR patients (n = 2). Additional information regarding damage to the native MV apparatus and the course of these patients is detailed in Supplemental Table S8. 

**Figure 1:**
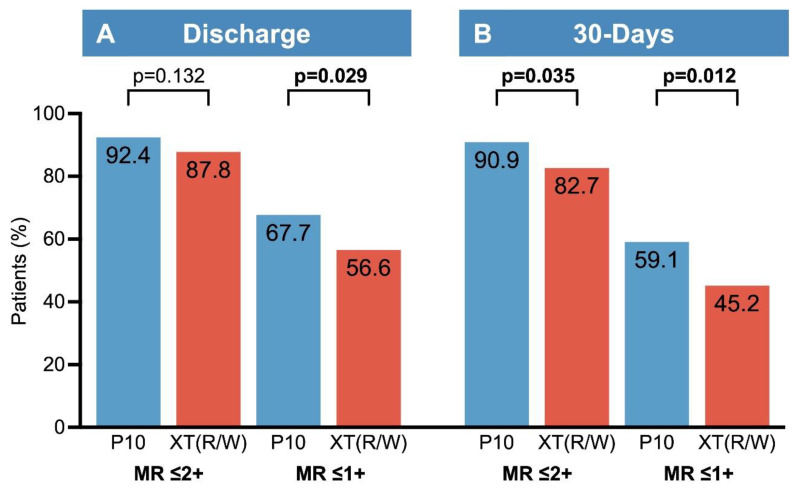
Reduction to MR ≤ 2+ and MR ≤ 1+ at discharge and 30 days. (**A**) The degree of MR ≤ 2+ at discharge was similar in both groups, although there were more patients with MR ≤ 1+ in the PASCAL group. (**B**) At 30-days, MR ≤ 2+ and ≤ 1+ were more frequently achieved in the PASCAL group. Patients with functional mitral regurgitation were the primary driver of the between-group differences. Abbreviations: FMR: Functional Mitral Regurgitation; MR: Mitral Regurgitation.

**Figure 2:**
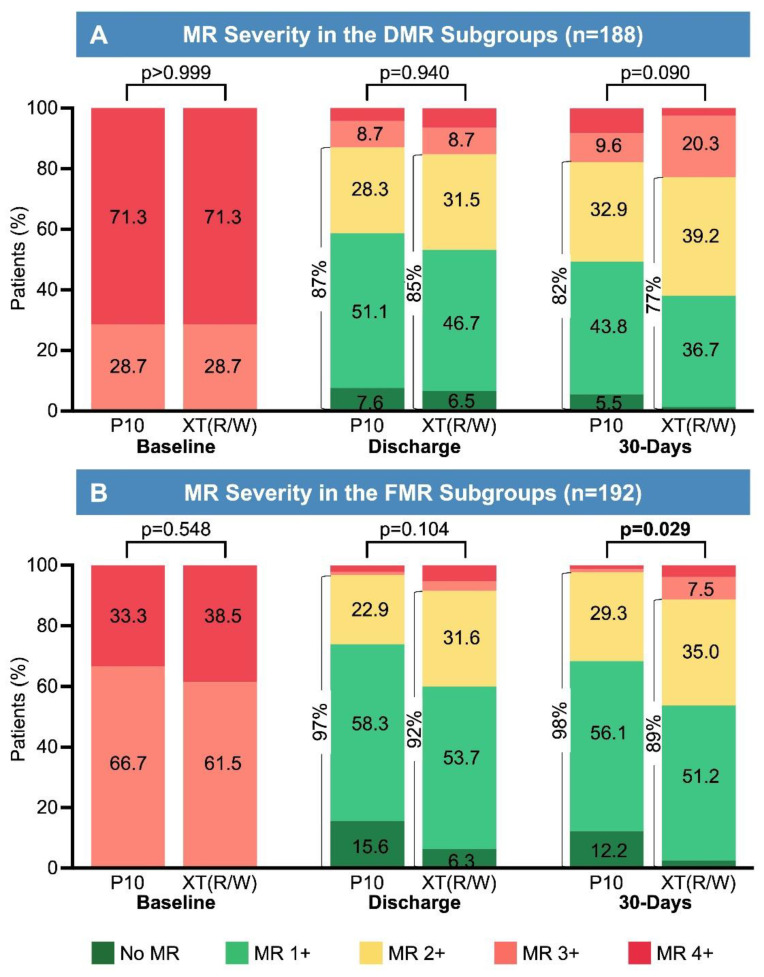
Reduction in MR severity according to MR etiology. (**A**) MR severity in the DMR subgroups. At discharge and 30 days, MR reduction to ≤ 2+ (*p* = 0.833 and *p* = 0.547) and ≤ 1+ (*p* = 0.553 and *p* = 0.191) was equally frequently achieved. (**B**) MR severity in the FMR subgroups. At discharge and 30-days, MR reduction to ≤ 2+ (*p* = 0.133 and *p* = 0.031) and ≤ 1+ (*p* = 0.046 and *p* = 0.076) tended to be achieved more frequently in patients treated with the PASCAL. The patients with mixed mitral regurgitation showed comparable results (Please refer to Supplemental Table S10). Abbreviations: DMR: Degenerative Mitral Regurgitation; FMR: Functional Mitral Regurgitation; MR: Mitral Regurgitation.

A significant MR reduction from baseline to discharge was achieved in PASCAL as well as in MitraClip patients (paired analysis *p* < 0.001 for both groups). At discharge, reduction to MR ≤ 2+ was achieved in 92.4% of PASCAL and 87.8% of MitraClip patients (*p* = 0.132), with reduction to MR ≤ 1+ in 67.7% of PASCAL and 56.6% of MitraClip patients (*p* = 0.029, Figure 1). The higher proportion of patients with a reduction to MR ≤ 1+ in the PASCAL group was driven by patients treated for FMR (PASCAL: 74.0% vs. MitraClip: 60.0%; *p* = 0.046), whereas results in DMR and MMR patients were comparable with both devices. Details of the MR etiology-based subgroup analysis are shown in Figure 2 and Supplemental Table S9. 

At discharge, the mean transmitral gradient rose from 2.0 (1.0–2.4) mm Hg at baseline to 3.0 (2.0–4.0) mm Hg in PASCAL patients (*p* < 0.001) and from 2.0 (1.4–2.7) mm Hg to 3.3 (2.4–4.3) mm Hg in MitraClip patients (*p* < 0.001). The median increase in the transmitral gradient was similar between the two groups (1.0 [0.0–2.0] mm Hg vs. 1.2 [0.5–2.1] mm Hg; *p* = 0.173). However, the mean transmitral gradient at discharge was higher in MitraClip (3.3 [2.4–4.3] mm Hg) than in PASCAL patients (3.0 [2.0–4.0] mm Hg; *p* = 0.038), Graphic Abstract. No differences in the MR etiology-based subgroups were observed, as shown in Supplemental Table S9.

Paired data on 3D-MVOA before and after the procedure were obtained for a subgroup of 65 PASCAL and 67 MitraClip patients. 3D-MVOA decreased from 4.5 (3.9–6.0) cm² at baseline to 3.0 (2.2–3.5) cm² at the end of the procedure in the PASCAL group (*p* < 0.001), and from 4.5 (3.9–5.4) cm² to 2.3 (1.0–2.9) cm² in the MitraClip group (*p* < 0.001). The median decrease in 3D-MVOA was significantly greater in the MitraClip than in the PASCAL group (PASCAL: 1.7 [1.0–3.4] cm² vs. MitraClip: 2.8 [1.9–3.8] cm²; *p* = 0.002). Consequently, the residual 3D-MVOA (PASCAL: 3.0 [2.2–3.5] cm² vs. MitraClip: 2.3 [1.0–2.9] cm²; *p* < 0.001) was significantly larger after PASCAL treatment (Graphic Abstract), and this difference persisted even after adjusting for baseline 3D-MVOA.

### 3.3. 30-Day Clinical Outcomes

Survival status at 30-days was available for 185 (92.5%) PASCAL and 187 (93.5%) MitraClip patients. The median follow-up period was 53 days (Q1-Q3: 30–97) for PASCAL and 84 days (Q1-Q3: 42–171) for MitraClip patients. Table 4 provides clinical outcomes up to 30-days. At 30-days, three patients in the PASCAL group and seven patients in the MitraClip group had died. Estimated 30-day mortality was comparable (PASCAL: 1.6% vs. MitraClip: 3.8%, log-rank *p* = 0.204).

NYHA-FC was obtained for 177 (88.5%) PASCAL and 170 (85.0%) MitraClip patients at 30-days. Both groups exhibited significant improvement in NYHA-FC (*p* < 0.001 compared to baseline). Patients treated with the PASCAL device showed a more pronounced reduction in NYHA-FC (*p* = 0.023). This difference was driven by more patients (n = 10) with NYHA-FC IV in the MitraClip group. Notably, the majority of these patients (n = 6) were treated for FMR, and two of these patients experienced periprocedural damage to the native MV apparatus, requiring subsequent reintervention. Consequently, the MR etiology-based subgroup analysis revealed differences in NYHA-FC reduction exclusively in FMR patients (Supplemental Table S10). No difference was observed in achieving NYHA-FC ≤ II (PASCAL: 73.4% vs. MitraClip: 72.9%, *p* > 0.999).

### 3.4. 30-Day Echocardiographic Outcomes 

Echocardiographic results up to 30-days are shown in Table 4 and were obtained for 164 (82.0%) PASCAL and 168 (84.0%) MitraClip patients. There were 4 cases of late SLDA observed in PASCAL and 2 in MitraClip patients. This resulted in a total of 6 and 4 patients with SLDA at 30-days in the PASCAL and MitraClip groups, respectively (*p* = 0.751). 

In terms of MR reduction, PASCAL patients showed a higher frequency of achieving reductions to both MR ≤ 2+ and MR ≤ 1+ at 30 days (Figure 1). In the MR etiology-based subgroup analysis, differences in MR reduction were observed foremost in patients treated for FMR (Supplemental Table S10 and Figure 2). The sustainability of MR reduction did not differ between PASCAL and MitraClip, nor within the MR etiology-based subgroups.

Device success was available for 174 (87.0%) PASCAL and 175 (87.5%) MitraClip patients, and no difference was observed between the two groups (PASCAL: 75.3% vs. MitraClip: 70.3%; *p* = 0.336), Table 4.

### 3.5. Sensitivity Analysis for MR Reduction

In a comparison between patients treated with PASCAL (n = 193) and MitraClip G3 (n = 147), there was no difference in overall MR (*p* = 0.235), MR ≤ 2+ (*p* = 0.183), and MR ≤ 1+ (*p* = 0.146) grade reduction at discharge. In contrast, at 30-days, MR grade reduction was more pronounced with PASCAL compared to MitraClip G3 (*p* = 0.006), which was not confirmed for achieving MR ≤ 2+ (*p* = 0.061) but was confirmed for achieving MR ≤ 1+ (*p* = 0.018). 

Comparing the PASCAL cohort (n = 193) with the MitraClip G4 cohort (n = 46), MR grade reduction at discharge was achieved more frequently with PASCAL than with MitraClip G4 (*p* = 0.002), which was confirmed for achieving MR ≤ 2+ (*p* = 0.017) and MR ≤ 1+ (*p* < 0.001). However, this was no longer observed at 30-days (reduction in MR grade overall: *p* = 0.121, MR ≤ 2+: *p* = 0.072, MR ≤ 1+: *p* = 0.089). 

A comparison between MitraClip G3 (n = 147) and G4 (n = 46) at discharge showed no difference in overall MR grade reduction (*p* = 0.142) or MR reduction to ≤ 2+ (*p* = 0.222). At discharge, however, MR reduction to ≤ 1+ was more frequently achieved with the MitraClip G3 vs. G4 (*p* = 0.024). No differences were found in MR reduction at 30-days (reduction in MR grade overall: *p* = 0.987, MR ≤ 2+: *p* = 0.808, MR ≤ 1+: *p* = 0.909).

## 4. Discussion

The current armamentarium of M-TEER devices, namely the PASCAL system with its P10 and Ace implants and the MitraClip system with its four different implant sizes, warrants a detailed comparison to guide individualized, patient-centered device selection in terms of efficacy and safety. This study presents the outcomes of a PS-matched cohort of 400 symptomatic MR patients treated with either the PASCAL P10 or G3 (XTR)/G4 (XT/XTW) MitraClip devices.

The main findings of our study are:

(1) Technical success rates were similarly high for both devices, at 96.5% for PASCAL and 96.0% for MitraClip patients.
(2) MR reduction to MR ≤ 2+ at discharge was comparable between PASCAL and MitraClip (92.4% vs. 87.8%). Reduction to MR ≤ 1+ was more frequently achieved in PASCAL patients (67.7% vs. 56.6%), primarily driven by the FMR subgroup.
(3) At 30 days, MR reduction to ≤ 2+ (90.9% vs. 82.7%) and ≤ 1+ (59.1% vs. 45.2%) was again more frequent in PASCAL patients, largely driven by the FMR subgroup.
(4) The mean transmitral gradient at discharge was higher in MitraClip patients (3.3 mm Hg vs. 3.0 mm Hg), accompanied by a smaller residual 3D-MVOA (2.3 cm² vs. 3.0 cm²).

To the best of our knowledge, this is the first and largest real-world, multicenter analysis comparing M-TEER outcomes of the large PASCAL P10 to the MitraClip XT(R/W). The PASCAL P10 and the MitraClip XT(R/W) devices have nearly equivalent span widths when open (26 mm and 25 mm, respectively) [2,4,19,20]. Despite design differences, both devices showed a similarly high technical success rate. Although the numbers were small, procedural damage to the native MV apparatus was observed only in patients treated with the MitraClip. This is consistent with previous reports of MV leaflet injury associated with the MitraClip XTR device and has been attributed to the increased tension on the MV leaflet exerted by the longer clip arms [19,20]. Even though previous studies were limited to the MitraClip G3 [19,20], it is likely that these results can be extrapolated to the G4, because only subtle changes have been made to the device itself, and we also observed damage to the native MV apparatus with the MitraClip G4. Although the low incidence of structural damage to the native MV apparatus precludes definitive conclusions, both present and previous data suggest very low rates of structural damage with the PASCAL system [19,20,21]. 

Independent grasping has the potential to lead to better procedural outcomes, as it can be utilized to optimize leaflet insertion. However, while this was introduced with the launch of the PASCAL, this feature was only introduced with the MitraClip G4. Interestingly, it was infrequently used with the MitraClip G4 not only in our study but also in a previous comparison and the EXPAND G4 study [4,9]. Why independent grasping with the MitraClip G4 is so infrequently used is not known. Nonetheless, the infrequent use of independent grasping and the results of our sensitivity analysis both suggest that our findings with MitraClip G3 are transferable to the MitraClip G4, even though only a quarter of MitraClip patients in our study were treated with the G4. 

Subtle, albeit statistically significant, differences in mean transmitral gradient at discharge were observed, with higher gradients in MitraClip patients. Consistent with this observation, the subset of patients with available 3D-MVOA data showed a significantly smaller residual 3D-MVOA in the MitraClip group. It might be speculated that the central spacer, the more flexible nitinol, and the fewer devices implanted may allow for a greater range of residual MV leaflet motion and subsequently a lower gradient in PASCAL patients. Although relevant mitral stenosis (mean transmitral gradient ≥ 5 mm Hg) did not occur more frequently in MitraClip patients, this difference may be considered for device selection in patients with a small baseline 3D-MVOA.

Recently, reduction to MR ≤ 1+ has been associated with superior survival, symptom relief, and reduced MR recurrence compared to patients with residual MR ≥ 2+ [22,23]. At discharge, both devices demonstrated a comparable reduction to MR ≤ 2+, a major measure of procedural efficacy. However, in patients treated with the PASCAL device, further reduction to MR ≤ 1+ was achieved more frequently. This observation persisted until 30-days, with PASCAL patients consistently achieving reductions to both MR ≤ 2+ and ≤ 1+ more frequently than MitraClip patients. The differences at 30-days can be largely explained by a minimally greater acute MR reduction and a slightly more sustained result in the PASCAL group. Interestingly, acute outcomes were comparable for DMR patients, and differences in MR reduction to MR ≤ 2+ and ≤ 1+ were solely driven by the FMR subgroup. It should be acknowledged that, despite PS-matching, the non-randomized nature of this study has inherent limitations due to unmeasured confounding factors, e.g., with respect to patient selection. Also, certain complexity parameters, such as single vs. multiple jets, jet width, central vs. commissural lesions, and calcifications, which have received increasing attention and may influence both procedural success and potentially device selection, were not included in this analysis [24]. Therefore, the results of the statistical comparison should merely be interpreted as hypothesis-generating.

Nevertheless, it can be speculated that some of the features of the PASCAL may have contributed to the observation of superior MR reduction in FMR patients. In these patients, who typically have a larger mitral annulus and thus a greater distance between the MV leaflets, the broad paddles and the central spacer of the PASCAL P10 may be advantageous, as both of these features address the relative leaflet deficit in FMR. In contrast, the MitraClip XT(R/W) achieves closer proximity to the MV leaflets; however, its narrower clip arms result in less leaflet tissue being captured, leaving a remaining coaptation gap on both sides. The ongoing CLASP-IIF trial (Edwards PASCAL TrAnScatheter Valve RePair System Pivotal Clinical Trial; NCT03706833) is enrolling FMR patients for randomization to therapy with either the PASCAL or the MitraClip, and this study could provide further insights; however, transferability to clinical practice may be limited due to the inclusion of a clinically and morphologically highly selected study population unrepresentative of current real-world practice. Contrary to our findings in FMR patients and consistent with the aforementioned hypothesis, no significant differences in MR reduction were observed within the DMR and MMR subgroups. The CLASP-IID trial reported largely comparable outcomes for DMR patients randomized to undergo M-TEER with either the PASCAL or the MitraClip [2]. Within this trial, a more pronounced reduction to MR ≤1+ with the PASCAL was observed; however, again, the strict clinical and morphological patient selection criteria might limit the transferability to daily clinical practice [2].

### Study Limitations

Although this is a rather large multicenter study with a wide range of interventionalists who performed the procedures as well as selected the devices, and we performed PS matching, which resulted in adequately balanced cohorts, selection bias related to individual device selection cannot be entirely excluded. This is particularly important as only three of the ten centers contributed patients to the MitraClip XT(R/W) cohort, and therefore operators and centers could be responsible for some of the outcome differences. In addition, detailed MV morphology was not available and was therefore not included in the PS matching. Moreover, the availability of the devices at any given time may have influenced the results. All echocardiographic and clinical parameters are site-reported and not independently adjudicated. To ascertain the quality of echocardiographic outcome measures, 20% of patients from the PASCAL cohort were reviewed by three echo experts from three centers (University Hospitals in Bonn, Cologne, and Duesseldorf), showing excellent agreement with the site-reported MR grading, as previously reported [11]. Echocardiographic outcomes in the MitraClip XT(R/W) cohort have been adjudicated by the same experts from the three centers providing MitraClip data. Furthermore, our study focused exclusively on the comparison between the PASCAL P10 and the large MitraClip XT(R/W), excluding the smaller PASCAL Ace and MitraClip NT. However, even in the most recently published studies, such as the CLASP-IID study and the PASCAL IID registry, patients were mainly treated with the PASCAL P10 [2,25]. Importantly, this study did not aim to compare the most contemporary generations of the two devices but rather to methodically analyze the impact of treatment with a large PASCAL device compared with a large MitraClip device. Finally, the lack of follow-up beyond 30-days limits the applicability of our findings to clinical practice.

## 5. Conclusions

M-TEER with the equally sized PASCAL P10 and the MitraClip XT(R/W) demonstrate high and comparable rates of technical success. Although an acute reduction to MR ≤ 2+ is achieved equally frequently with both devices, treatment with the PASCAL seems to be favorable in achieving a reduction to MR ≤ 1+ in patients treated for FMR.

## Figures and Tables

**Table 1:** Baseline characteristics.

	PASCAL P10	MitraClip XT(R/W)	*p*-Value
	n = 200	n = 200	
Age, years	80 (73–84)	79 (73–83)	0.548
Female	91 (45.5)	101 (50.5)	0.368
NYHA functional class			0.970
II	32 (16.0)	31 (15.5)	
III	150 (75.0)	152 (76.0)	
IV	18 (9.0)	17 (8.5)	
NTproBNP, ng/L *	2330 (1246–4735)	2342 (1280–4750)	0.849
EuroSCORE II, %	4.3 (2.9–6.5)	4.1 (2.5–6.8)	0.643
Comorbidities			
Arterial hypertension	176 (88.0)	148 (74.0)	<0.001
Diabetes mellitus	51 (25.5)	30 (15.0)	0.013
Coronary artery disease	102 (51.0)	104 (52.0)	0.920
Previous myocardial infarction	31 (15.5)	32 (16.0)	>0.999
Previous cardiac surgery	35 (17.5)	36 (18.0)	>0.999
ICD/CRT	35 (17.5)	43 (21.5)	0.377
Atrial fibrillation	139 (69.5)	151 (75.5)	0.218
Chronic lung disease	43 (21.5)	24 (12.0)	0.016
eGFR, mL/min	49 (35–66)	45 (34–62)	0.328
Chronic renal disease (eGFR < 60 mL/min)	135 (67.5)	140 (70.7)	0.516
On dialysis	2 (1.0)	6 (3.0)	0.284

Values are n (%) or median (Q1–Q3). CRT: Cardiac Resynchronization Therapy; eGFR: estimated Glomerular Filtration Rate (calculated using the Cockcroft–Gault equation); ICD: Implantable Cardioverter Defibrillator; NYHA: New York Heart Association. * Pre-procedural NTproBNP data were obtained for a subgroup of 153 PASCAL patients and 177 MitraClip patients.

**Table 2:** Baseline echocardiographic characteristics.

	PASCAL P10	MitraClip XT(R/W)	*p*-Value
	n = 200	n = 200	
MR etiology			> 0.999
Degenerative MR	94 (47.0)	94 (47.0)	
Functional MR	96 (48.0)	96 (48.0)	
Mixed MR	10 (5.0)	10 (5.0)	
MR severity			
Effective regurgitant orifice area, mm²	34 (25–49)	35 (27–48)	0.834
Left ventricle			
Left ventricular ejection fraction, %	54 (39–60)	55 (42–61)	0.509
Left ventricular end-diastolic diameter, mm	55 (49–61)	54 (50–61)	0.779
Left ventricular end-systolic diameter, mm	39 (33–50)	39 (31–48)	0.219
Pre-procedural 3D mitral valve orifice area, cm² *	4.5 (3.9–6.0)	4.5 (3.9–5.4)	0.265
Mean transmitral gradient, mm Hg	2.0 (1.0–2.4)	2.0 (1.4–2.7)	0.232
Left atrial volume index, mL/m²	62 (49–83)	59 (45–77)	0.090
Right ventricle			
Degree of TR			0.084
No TR	1 (0.5)	7 (3.5)	
Mild TR	76 (38.2)	64 (32.3)	
Moderate TR	73 (36.7)	68 (34.3)	
Severe TR	49 (24.6)	59 (29.8)	
Systolic pulmonary artery pressure, mm Hg	45 (35–55)	46 (38–58)	0.223

Values are n (%) or median (Q1–Q3). 3D: 3-Dimensional; MR: Mitral Regurgitation; TR: Tricuspid Regurgitation. * Pre-procedural 3D mitral valve orifice data were obtained for a subgroup of 129 PASCAL patients and 196 MitraClip patients.

**Table 3:** Procedural and in-hospital outcomes.

	PASCAL P10	MitraClip XT(R/W)	*p*-Value
	n = 200	n = 200	
Technical success *	193 (96.5)	192 (96.0)	> 0.999
Procedural mortality	0 (0.0)	0 (0.0)	
Procedure aborted	5 (2.5)	4 (2.0)	> 0.999
Procedural single leaflet device attachment	2 (1.0)	2 (1.0)	> 0.999
Damage to the native mitral valve apparatus	0 (0.0)	4 (2.0)	0.123
Conversion to mitral valve surgery	0 (0.0)	1 (0.5)	> 0.999
Devices implanted			< 0.001
0	5 (2.5)	4 (2.0)	
1	142 (71.0)	84 (42.0)	
2	53 (26.5)	98 (49.0)	
3	0 (0.0)	14 (7.0)	
Degree of residual MR (n = 394/400)			0.067
No MR	25 (12.6)	12 (6.1)	
1+	109 (55.1)	99 (50.5)	
2+	49 (24.7)	61 (31.1)	
3+	9 (4.5)	12 (6.1)	
4+	6 (3.0)	12 (6.1)	
MR ≤ 2+	183 (92.4)	172 (87.8)	0.132
MR ≤ 1+	134 (67.7)	111 (56.6)	0.029
Mean transmitral gradient, mm Hg	3.0 (2.0–4.0)	3.3 (2.4–4.3)	0.038
Increase in mean transmitral gradient, mm Hg	1.0 (0.0–2.0)	1.2 (0.5–2.1)	0.173
Mean transmitral gradient ≥ 5 mm Hg	25 (16.2)	28 (14.3)	0.654
Residual 3D mitral valve orifice area, cm² †	3.0 (2.2–3.5)	2.3 (1.0–2.9)	< 0.001
Procedure duration, minutes	83 (60–117)	86 (62–119)	0.783

Values are n (%) or median (Q1–Q3). 3D: 3-Dimensional; MR: Mitral Regurgitation.* More than one criterion for lack of technical success could apply. † Data on residual mitral valve orifice area was obtained for a subgroup of 68 PASCAL and 69 MitraClip patients.

**Table 4:** Follow-up outcomes at 30 days.

	PASCAL P10	MitraClip XT(R/W)	*p*-Value
	n = 200	n = 200	
All-cause mortality (n = 368/400)	3 (1.6)	7 (3.8)	0.337
Severe bleeding	6 (3.0)	9 (4.6)	0.443
Stroke	1 (0.5)	2 (1.0)	0.621
Renal failure requiring dialysis	1 (0.5)	3 (1.5)	0.369
Single leaflet device attachment	6 (3.0)	4 (2.1)	0.751
Reintervention for mitral valve dysfunction	1 (0.5)	4 (2.0)	0.213
NYHA functional class (n = 347)			0.023
I	47 (26.6)	32 (18.8)	
II	83 (46.9)	92 (54.1)	
III	45 (25.4)	36 (21.2)	
IV	2 (1.1)	10 (5.9)	
MR severity (n = 332)			0.002
No MR	15 (9.1)	3 (1.8)	
1+	82 (50.0)	73 (43.5)	
2+	52 (31.7)	63 (37.5)	
3+	8 (4.9)	23 (13.7)	
4+	7 (4.3)	6 (7.3)	
MR ≤ 2+	149 (90.9)	139 (82.7)	0.035
MR ≤ 1+	97 (59.1)	76 (45.2)	0.012
Mean transmitral gradient, mm Hg	3.0 (2.4–4.0)	3.2 (2.6–4.3)	0.217
Increase in mean transmitral gradient, mm Hg	1.1 (0.8–2.0)	1.3 (0.5–2.3)	0.892
Mean transmitral gradient ≥ 5 mm Hg	23 (11.6)	23 (11.5)	> 0.999
Device success (n = 349)	131 (75.3)	123 (70.3)	0.336

Values are n (%) or median (Q1–Q3). Abbreviations: MR: Mitral Regurgitation NYHA: New York Heart Association.

## Data Availability

The data that support the findings of this study are available from the corresponding author upon reasonable request.

## Supplementary Materials

The following supporting information can be downloaded at: https://www.mdpi.com/article/10.3390/jcm13144187/s1, Figure S1: Standardized mean Differences before and after Matching within Mitral Regurgitation Etiologies; Table S1: Baseline Characteristics before Matching; Table S2: Echocardiographic Assessment at Baseline before Matching; Table S3: Procedural Outcomes of the MitraClip cohort before Matching; Table S4: 30-day Outcomes of the MitraClip Cohort before Matching; Table S5: Baseline Characteristics of MR Etiologies after Matching; Table S6: Echocardiographic Assessment of MR Etiologies at Baseline after Matching; Table S7: MitraClip Combinations; Table S8: Damage to the Native Mitral Valve Apparatus; Table S9: Procedural Outcomes of MR Etiologies after Matching; Table S10: 30-day Outcomes of MR Etiologies after Matching.
