# Supplementary material for: Early Outcomes of Two Large Mitral Valve Transcatheter Edge-to-Edge Repair Devices—A Propensity Score Matched Multicenter Comparison
Source: J Clin Med. 2024 Jul 17;13(14):4187. doi: 10.3390/jcm13144187 (PMC11278441; doi:10.3390/jcm13144187)
Supplement: Supplementary file 1 [file jcm-13-04187-s001.zip › jcm-3065927-supplementary.pdf]

Supplemental Material

Supplemental Figure S1: Standardized mean Differences before and after Matching within Mitral Regurgitation Etiologies

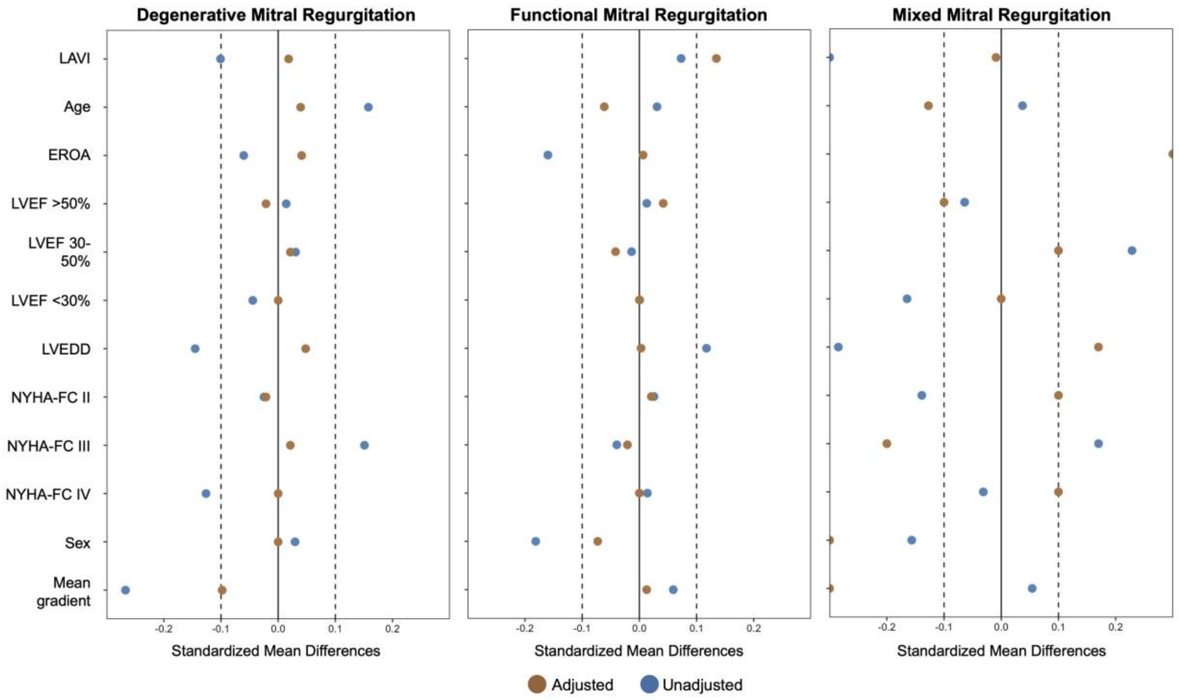

Abbreviations: EROA: Effective Regurgitant Orifice Area, LAVI: Left Atrial Volume Index, LVEDD: Left Ventricular End-Diastolic Diameter, LVEF: Left Ventricular Ejection Fraction, NYHA-FC: New York Heart Association Functional Class.

Supplemental Table S1: Baseline Characteristics before Matching

**Characteristic**	MitraClip XT(R/W)
	n=253
Age, years	79 (73 – 83)
Female	130 (51.4)
NYHA functional class	
II	42 (16.6)
III	175 (69.2)
IV	36 (14.2)
NTproBNP, ng/L*	2321 (1164 – 4759)
EuroSCORE II, %	4.6 (2.6 – 7.1)
Comorbidities	
Arterial hypertension	191 (75.5)
Diabetes mellitus	39 (15.4)
Coronary artery disease	124 (49.0)
Previous myocardial infarction	36 (14.2)
Previous cardiac surgery	44 (17.4)
ICD/CRT	48 (19.0)
Atrial fibrillation	190 (75.1)
Chronic lung disease	34 (13.4)
eGFR, ml/min	45 (34 – 62)
Renal disease (eGFR <60 ml/min)	179 (71.6)
On dialysis	7 (2.8)

Values are n (%), mean±SD or median (Q1 – Q3). CRT: Cardiac Resynchronization Therapy, eGFR: estimated Glomerular Filtration Rate (calculated using the Cockcroft–Gault equation), ICD: Implantable Cardioverter Defibrillator, NYHA: New York Heart Association. *Data on pre-procedural NTproBNP were available for a subgroup of 220 PASCAL and 219 MitraClip patients, respectively.

Supplemental Table S2: Echocardiographic Assessment at Baseline before Matching

Characteristic	MitraClip XT(R/W)
	n=253
MR etiology	
Degenerative MR	135 (53.4)
Functional MR	103 (40.7)
Mixed MR	15 (5.9)
MR severity	
3+	97 (38.3)
4+	156 (61.7)
Effective regurgitant orifice area, mm^2	38 (29 – 53)
Left ventricle	
Left ventricular ejection fraction, %	55 (44 – 61)
Left ventricular end-diastolic diameter, mm	55 (50 – 62)
Left ventricular end-systolic diameter, mm	39 (31 – 48)
Left ventricular end-diastolic volume, ml	125 (95 – 169)
Left ventricular end-systolic volume, ml	57 (37 – 90)
Pre-procedural 3D mitral valve orifice area, cm^2 **	4.4 (3.8 – 5.5)
Mean transmitral gradient, mm Hg	2.0 (1.4 – 2.9)
Left atrial volume index, ml/m^2	60 (45 – 80)
Right ventricle	
TR severity	
No TR	7 (2.8)
Mild TR	83 (33.5)
Moderate TR	88 (35.5)
Severe TR	70 (28.2)
Systolic pulmonary artery pressure, mm Hg	47 (38 – 58)

Values are median (Q1 – Q3) or n (%). Abbreviations: 3D: 3-Dimensional, MR: Mitral Regurgitation, TR: Tricuspid Regurgitation. *Data on pre-procedural 3D mitral valve orifice area were available for a subgroup of 197 PASCAL and 247 MitraClip patients, respectively.

Supplemental Table S3: Procedural Outcomes of the MitraClip cohort before Matching

**Procedural Outcomes**	MitraClip XT(R/W)
Technical success	242 (95.7)
Intraprocedural single-leaflet device attachment	2 (0.8)
Procedure aborted	9 (3.6)
Procedural death	0 (0.0)
Conversion to open heart surgery	1 (0.4)
Damage to the native mitral valve apparatus	7 (2.8)
Devices implanted	
0	9 (3.6)
1	111 (43.9)
2	116 (45.8)
3	16 (6.3)
4	1 (0.4)
Degree of mitral regurgitation at discharge (n=246)	
0+	19 (7.7)
1+	118 (48.0)
2+	77 (31.3)
3+	14 (5.7)
4+	18 (7.3)
Mean transmitral gradient at discharge, mm Hg	3.2 (2.4 – 4.3)
Procedure duration (min)	87 (62 – 120)

Values are n (%) or mean ± SD.

Supplemental Table S4: 30-day Outcomes of the MitraClip Cohort before Matching

**30-day Outcomes**	MitraClip XT(R/W)
All-cause mortality at 30-days (n=240)	10 (4.2)
Single-leaflet device attachment	9 (3.7)
Reintervention for device failure	4 (1.6)
Severe bleeding	12 (4.9)
Cerebrovascular event	3 (1.2)
Renal failure requiring dialysis	4 (1.6)
NYHA functional class (n=210)	
I	39 (18.6)
II	115 (54.8)
III	42 (20.0)
IV	14 (6.7)
Mitral regurgitation severity (n=204)	
No MR	5 (2.5)
1+	88 (43.1)
2+	75 (36.8)
3+	28 (13.7)
4+	8 (3.9)
MR ≤2+	168 (82.4)
MR ≤1+	93 (45.6)
Mean transmitral gradient, mm Hg	3.2 (2.7 – 4.3)
Mean transmitral gradient ≥5 mm Hg	28 (14.6)
Device success (n=238)	161 (67.6)

Values are n (%) or mean ± SD.

Supplemental Table S5: Baseline Characteristics of MR Etiologies after Matching

	Degenerative MR			Functional MR			Mixed MR		
	PASCAL P10	MitraClip XT(R/W)	p-value	PASCAL P10	MitraClip XT(R/W)	p-value	PASCAL P10	MitraClip XT(R/W)	p-value
	n=94	n=94		n=96	n=96		n=10	n=10	
Age, years	82 (79 – 85)	81 (77 – 84)	0.255	77 (69 – 81)	76 (69 – 81)	0.974	78 (73 – 83)	79 (73 – 83)	0.970
Female	48 (51.1)	48 (51.1)	0.999	39 (40.6)	46 (47.9)	0.383	4 (40.0)	7 (70.0)	0.370
NYHA-FC			0.963			0.969			0.628
II	17 (18.1)	19 (20.2)		14 (14.6)	12 (12.5)		1 (10.0)	0 (0.0)	
III	72 (76.6)	70 (74.5)		72 (75.0)	74 (77.1)		6 (60.0)	8 (80.0)	
IV	5 (5.3)	5 (5.3)		10 (10.4)	10 (10.4)		3 (30.0)	2 (20.0)	
NTproBNP, ng/L	1652 (791 – 3290)	1645 (788 – 3433)	0.884	3485 (1839 – 5094)	2927 (1573 – 6800)	0.688	3823 (1210 – 10949)	2503 (2063 – 5707)	0.779
EuroSCORE II, %	4.3 (3.0 – 6.2)	3.8 (2.3 – 5.0)	**0.011**	4.3 (3.0 – 6.9)	5.4 (2.6 – 10.9)	0.190	3.6 (2.3 – 6.7)	5.4 (4.4 – 9.1)	0.481
Comorbidities									
Arterial hypertension	81 (86.2)	72 (76.6)	0.133	87 (90.6)	69 (71.9)	**0.001**	8 (80.0)	7 (70.0)	0.999
Diabetes mellitus	20 (21.3)	7 (7.4)	**0.011**	30 (31.3)	21 (21.9)	0.191	1 (10.0)	2 (20.0)	0.999
Coronary artery disease	46 (48.9)	36 (38.3)	0.185	53 (55.2)	61 (63.5)	0.304	3 (30.0)	7 (70.0)	0.179
Previous MI	7 (7.4)	2 (2.1)	0.169	23 (24.0)	27 (28.1)	0.622	1 (10.0)	3 (30.0)	0.582
Previous cardiac surgery	17 (18.1)	8 (8.5)	0.084	16 (16.7)	26 (27.1)	0.115	2 (20.0)	2 (20.0)	0.999
ICD/CRT	7 (7.4)	3 (3.2)	0.330	26 (27.1)	37 (38.5)	0.124	2 (20.0)	3 (30.0)	0.999
Atrial fibrillation	64 (68.1)	68 (72.3)	0.633	68 (70.8)	76 (79.2)	0.243	7 (70.0)	7 (70.0)	0.999
Chronic lung disease	20 (21.3)	10 (10.6)	0.072	19 (19.8)	14 (14.6)	0.445	4 (40.0)	0 (0.0)	0.087
eGFR, ml/min	49 (38 – 60)	46 (38 – 62)	0.883	54 (32 – 73)	45 (32 – 64)	0.322	50 (35 – 56)	33 (25 – 39)	0.218
Renal disease (eGFR <60 ml/min)	70 (74.5)	66 (71.7)	0.742	57 (59.4)	65 (67.7)	0.294	8 (80.0)	9 (90.0)	0.999
On dialysis	1 (1.1)	1 (1.1)	0.999	1 (1.0)	4 (4.2)	0.368	0 (0.0)	1 (10.0)	0.999

Values are n (%), mean±SD or median (Q1 – Q3). Abbreviations: CRT: Cardiac Resynchronization Therapy, eGFR: estimated Glomerular Filtration Rate (calculated using the Cockcroft–Gault equation), ICD: Implantable Cardioverter Defibrillator, NYHA-FC: New York Heart Association Functional Class.

Supplemental Table S6: Echocardiographic Assessment of MR Etiologies at Baseline after Matching

	Degenerative MR			Functional MR			Mixed MR		
	PASCAL P10	MitraClip XT(R/W)	p-value	PASCAL P10	MitraClip XT(R/W)	p-value	PASCAL P10	MitraClip XT(R/W)	p-value
	n=94	n=94		n=96	n=96		n=10	n=10	
MR severity			0.999			0.548			0.999
3+	27 (28.7)	27 (28.7)		64 (66.7)	59 (61.5)		6 (60.0)	7 (70.0)	
4+	67 (71.3)	67 (71.3)		32 (33.3)	37 (38.5)		4 (40.0)	3 (30.0)	
EROA, mm²	47 (31 – 65)	45 (31 – 60)	0.578	30 (22 – 39)	32 (24 – 40)	0.233	31 (25 – 39)	31 (21 – 34)	0.570
Left ventricle									
LVEF, %	58 ± 8	59 ± 8	0.117	41 (30 – 56)	42 (29 – 55)	0.870	47 ± 12	49 ± 15	0.707
LVEDD, mm	52 ± 7	51 ± 8	0.755	60 ± 10	60 ± 11	0.999	57 ± 10	54 ± 4	0.458
LVESD, mm	36 ± 8	34 ± 8	0.125	46 (37 – 57)	45 (38 – 55)	0.817	39 (38 – 43)	30 (28 – 44)	0.229
LVEDV, ml	110 (85 – 131)	111 (89 – 134)	0.958	154 (94 – 194)	148 (116 – 196)	0.587	135 ± 65	114 ± 48	0.423
LVESV, ml	44 (35 – 58)	43 (34 – 58)	0.511	86 (43 – 135)	82 (56 – 130)	0.855	88 ± 58	61 ± 38	0.245
Mean transmitral gradient, mm Hg	2.0 (1.0 – 3.0)	2.1 (1.4 – 3.0)	0.139	2.0 (1.0 – 2.0)	1.7 (1.1 – 2.2)	0.717	2.0 (1.0 – 2.0)	2.0 (1.7 – 3.0)	0.356
LAVI, ml/m²	58 (46 – 79)	57 (43 – 73)	0.377	67 (52 – 87)	62 (46 – 80)	0.127	70 ± 17	70 ± 34	0.979
Right ventricle									
TR severity			0.431			0.217			0.097
No TR	1 (1.1)	3 (3.3)		0 (0.0)	2 (2.1)		0 (0.0)	2 (20.0)	
Mild TR	42 (45.2)	32 (34.8)		28 (29.2)	31 (32.3)		6 (60.0)	1 (10.0)	
Moderate TR	34 (36.6)	38 (41.3)		38 (39.6)	27 (28.1)		1 (10.0)	3 (30.0)	
Severe TR	16 (17.2)	19 (20.7)		30 (31.3)	36 (37.5)		3 (30.0)	4 (40.0)	
sPAP, mm Hg	46 (38 – 58)	46 (39 – 60)	0.707	45 ± 14	46 ± 13	0.604	36 ± 9	47 ± 9	**0.017**

Values are n (%) or mean ±SD. Abbreviations: EROA: Effective Regurgitant Orifice Area, LAVI: Left Atrial Volume Index, LVEDD: Left Ventricular End-Diastolic Diameter, LVEDV: Left Ventricular End-Diastolic Volume, LVEF: Left Ventricular Ejection Fraction, LVESD: Left Ventricular End-Systolic Diameter, LVESV: Left Ventricular End-Systolic Volume, MR: Mitral Regurgitation, sPAP: systolic Pulmonary Artery Pressure, TR: Tricuspid Regurgitation.

Supplemental Table S7: MitraClip Combinations

**Device**	**MitraClip XT(R/W)**
	n=200
G3 MitraClip XTR	92 (46.0)
G3 MitraClip XTR+NTR	58 (29.0)
G3 MitraClip XTR + G4 MitraClip XT	1 (0.5)
G4 MitraClip XT	14 (7.0)
G4 MitraClip XTW	17 (8.5)
G4 MitraClip XT+NTW	1 (0.5)
G4 MitraClip XT+XTW	2 (1.0)
G4 MitraClip XT+XTW+NT	1 (0.5)
G4 MitraClip XT+NTW+NT	1 (0.5)
G4 MitraClip XTW+NT	6 (3.0)
G4 MitraClip XTW+NTW	1 (0.5)
G4 MitraClip XTW+NTW+NT	2 (1.0)

Supplemental Table S8: Damage to the Native Mitral Valve Apparatus

Damage to the native mitral valve apparatus (n=4)	
#1 (MitraClip), DMR	The patient was intended to be treated with a MitraClip XTW for degenerative MR. During the procedure, detachment of the MitraClip XTW from the posterior mitral leaflet was observed. A second device (MitraClip XT) was positioned next to the MitraClip XTW. After releasing the MitraClip XT, a detachment of the anterior mitral leaflet and injury of the anterior mitral leaflet were observed. The patient underwent MV replacement 100 days after the index procedure.
#2 (MitraClip), DMR	The patient was intended to be treated with a MitraClip XTR for degenerative MR. During the procedure the posterior mitral leaflet was injured with the MitraClip XTR, resulting in progression of MR that rapidly led to cardiogenic shock, so conversion to MV replacement was performed, which included closure of the iatrogenic atrial septal defect and repair of the tricuspid valve. Progressive circulatory instability ensued and extracorporeal life support was initiated, but the patient eventually died of intracerebral hemorrhage during the index-hospitalization.
#3 (MitraClip), FMR	The patient was intended to be treated with a MitraClip XTR for functional MR. During the procedure the MitraClip XTR was intended to be re-positioned, after this maneuver the anterior mitral leaflet was injured leading to progression of MR. After that, attempts were made for some time to grasp the leaflets with the XTR, but no sufficient MR reduction was achieved so the procedure was aborted without implantation of any device. The patient underwent reintervention for MR 132 days after the index-procedure.
#4 (MitraClip), FMR	The patient was intended to be treated with two MitraClips XTR for functional MR. The first MitraClip XTR could reduce MR sufficiently but the second MitraClip caused a perforation of the posterior mitral leaflet, leading to progression of MR. During follow-up PASCAL implantation was performed.

Supplemental Table S9: Procedural Outcomes of MR Etiologies after Matching

	Degenerative MR			Functional MR			Mixed MR		
	PASCAL P10	MitraClip XT(R/W)	p-value	PASCAL P10	MitraClip XT(R/W)	p-value	PASCAL P10	MitraClip XT(R/W)	p-value
	n=94	n=94		n=96	n=96		n=10	n=10	
Technical success	90 (95.7)	91 (96.8)	0.999	93 (96.9)	92 (94.8)	0.721	10 (100)	10 (100)	
Procedural death	0 (0.0)	0 (0.0)		0 (0.0)	0 (0.0)		0 (0.0)	0 (0.0)	
Procedure aborted	2 (2.1)	1 (1.1)	0.999	3 (3.1)	3 (3.1)	0.999	0 (0.0)	0 (0.0)	
Procedural SLDA	2 (2.1)	1 (1.1)	0.999	0 (0.0)	1 (1.0)	0.999	0 (0.0)	0 (0.0)	
Damage to the native MV apparatus	0 (0.0)	2 (2.1)	0.497	0 (0.0)	2 (2.1)	0.497	0 (0.0)	0 (0.0)	
Conversion to open heart surgery	0 (0.0)	1 (1.1)	0.999	0 (0.0)	0 (0.0)		0 (0.0)	0 (0.0)	
Devices implanted			**<0.001**			**<0.001**			0.999
0	2 (2.1)	1 (1.1)		3 (3.1)	3 (3.1)		0 (0.0)	0 (0.0)	
1	68 (72.3)	39 (41.5)		68 (70.8)	39 (40.6)		6 (60.0)	6 (60.0)	
2	24 (25.5)	47 (50.0)		25 (26.0)	47 (49.0)		4 (40.0)	4 (40.0)	
3	0 (0.0)	7 (7.4)		0 (0.0)	7 /7.3)		0 (0.0)	0 (0.0)	
MR severity at discharge			0.940			0.104			0.373
No MR	7 (7.6)	6 (6.5)		15 (15.6)	6 (6.3)		3 (30.0)	0 (0.0)	
1+	47 (51.1)	43 (46.7)		56 (58.3)	51 (53.7)		6 (60.0)	5 (55.6)	
2+	26 (28.3)	29 (31.5)		22 (22.9)	30 (31.6)		1 (10.0)	2 (22.2)	
3+	8 (8.7)	8 (8.7)		1 (1.0)	3 (3.2)		0 (0.0)	1 (11.1)	
4+	4 (4.3)	6 (6.5)		2 (2.1)	5 (5.3)		0 (0.0)	1 (11.1)	
MR ≤ 2+	80 (87.0)	78 (84.8)	0.833	93 (96.9)	87 (91.6)	0.133	10 (100)	7 (77.8)	0.211
MR ≤ 1+	54 (58.7)	49 (53.3)	0.553	71 (74.0)	57 (60.0)	**0.046**	9 (90.0)	5 (55.6)	0.141
Mean transmitral gradient at discharge, mm Hg	3.0 (2.0 – 4.0)	3.6 (2.6 -4.4)	0.155	3.0 (2.0 – 4.0)	3.0 (2.2 – 4.0)	0.174	2.3 ± 1.2	3.4 ± 1.7	0.198
Increase in mean transmitral gradient at discharge, mm Hg	1.2 ± 1.4	1.3 ± 1.4	0.571	1.0 (0.1 – 2.0)	1.2 (0.7 – 2.1)	0.197	0.7 ± 1.4	1.2 ± 1.2	0.431

Mean transmitral gradient ≥5 mmHg at discharge, mm Hg	16 (20.8)	14 (15.2)	0.420	9 (12.7)	12 (12.6)	0.999	0 (0.0)	2 (22.2)	0.486
Procedure duration, minutes	88 (60 – 123)	81 (63 – 121)	0.788	80 (58 – 103)	90 (60 – 111)	0.427	109 ± 59	101 ± 38	0.717

Values are n (%) or mean±SD. Abbreviations: MR: Mitral Regurgitation, MV: Mitral Valve, SLDA: Single-Leaflet Device Attachment.

Supplemental Table S10: 30-day Outcomes of MR Etiologies after Matching

	Degenerative MR			Functional MR			Mixed MR		
	PASCAL P10	MitraClip XT(R/W)	p-value	PASCAL P10	MitraClip XT(R/W)	p-value	PASCAL P10	MitraClip XT(R/W)	p-value
	n=94	n=94		n=96	n=96		n=10	n=10	
All-cause mortality	1 (1.1)	3 (3.4)	0.621	2 (2.3)	3 (3.3)	0.999	0 (0.0)	1 (11.1)	0.999
Severe bleeding	2 (2.1)	3 (3.3)	0.681	4 (4.2)	6 (6.3)	0.537	0 (0.0)	0 (0.0)	
Stroke	1 (1.1)	1 (1.1)	0.999	0 (0.0)	1 (1.1)	0.497	0 (0.0)	0 (0.0)	
Renal failure requiring dialysis	1 (1.1)	1 (1.1)	0.999	0 (0.0)	1 (1.1)	0.497	0 (0.0)	1 (10.0)	0.999
SLDA	6 (6.6)	3 (3.3)	0.497	0 (0.0)	1 (1.0)	0.492	0 (0.0)	0 (0.0)	
Reintervention for MV dysfunction	1 (1.1)	3 (3.3)	0.366	0 (0.0)	1 (1.0)	0.497	0 (0.0)	0 (0.0)	
NYHA functional class			0.392			**0.017**			0.896
I	22 (27.2)	22 (27.8)		22 (25.3)	9 (11.0)		3 (33.3)	1 (11.1)	
II	42 (51.9)	41 (51.9)		38 (43.7)	46 (56.1)		3 (33.3)	5 (55.6)	
III	17 (21.0)	13 (16.5)		26 (29.9)	21 (25.6)		2 (22.2)	2 (22.2)	
IV	0 (0.0)	3 (3.8)		1 (1.1)	6 (7.3)		1 (11.1)	1 (11.1)	
MR severity			0.090			**0.029**			0.999
No MR	4 (5.5)	1 (1.3)		10 (12.2)	2 (2.5)		1 (11.1)	0 (0.0)	
1+	32 (43.8)	29 (36.7)		46 (56.1)	41 (51.2)		4 (44.4)	3 (33.3)	
2+	24 (32.9)	31 (39.2)		24 (29.3)	28 (35.0)		4 (44.4)	4 (44.4)	
3+	7 (9.6)	16 (20.3)		1 (1.2)	6 (7.5)		0 (0.0)	1 (11.1)	
4+	6 (8.2)	2 (2.5)		1 (1.2)	3 (3.8)		0 (0.0)	1 (11.1)	
MR ≤2+	60 (82.2)	61 (77.2)	0.547	80 (97.6)	71 (88.8)	**0.031**	9 (100)	7 (77.8)	0.471
MR ≤1+	36 (49.3)	30 (38.0)	0.191	56 (68.3)	43 (53.8)	0.076	5 (55.6)	3 (33.3)	0.637
Mean transmitral gradient, mm Hg	3.0 (2.7 – 4.7)	3.7 (2.6 – 4.3)	0.830	3.0 (2.2 – 3.0)	3.6 (2.6 – 4.4)	0.274	3.0 (2.2 – 3.0)	3.6 (2.6 – 4.4)	0.370
Mean transmitral gradient ≥5 mm Hg	17 (18.3)	14 (14.9)	0.561	5 (5.2)	8 (8.3)	0.567	1 (10.0)	1 (10.0)	0.999
Device success	48 (60.8)	52 (63.4)	0.748	75 (87.2)	65 (78.3)	0.154	8 (88.9)	6 (60.0)	0.303

Values are n (%) or mean ±SD. Abbreviations: MR: Mitral Regurgitation, MV: Mitral Valve, NYHA: New York Heart Association, SLDA: Single Leaflet Device Attachment.
